# Interleukin-17A promotes functional activation of systemic sclerosis patient-derived dermal vascular smooth muscle cells by extracellular-regulated protein kinases signalling pathway

**DOI:** 10.1186/s13075-014-0512-2

**Published:** 2014-12-31

**Authors:** Mengguo Liu, Ji Yang, Xiaojing Xing, Xiangxiang Cui, Ming Li

**Affiliations:** Department of Dermatology, Zhongshan Hospital, Fudan University, 180 Fenglin Road, Shanghai, 200032 China

## Abstract

**Introduction:**

Dermal vascular smooth muscle cells (DVSMCs) are important for vascular wall fibrosis in microangiopathy of systemic sclerosis (SSc). T helper 17 cell-associated cytokines, particularly interleukin-17A (IL-17A), have been demonstrated to play a role in the pathogenesis of SSc. However, the effect of IL-17A on the DVSMCs in microangiopathy of SSc has not been established. In the present study, we investigated the effect of IL-17A on the SSc patient-derived DVSMCs.

**Methods:**

DVSMCs from patients with SSc and healthy subjects were incubated using IL-17A or serum derived from patients with SSc. Subsequently, the proliferation, collagen synthesis and secretion, and migration of DVSMCs were analysed using a cell counting kit-8 (CCK-8), dual-luciferase reporter assay, real-time reverse transcription-polymerase chain reaction (RT-PCR), Western blot, enzyme-linked immunosorbent assay (ELISA) and transwell assay. The protein phosphorylation of signalling pathways in the process of IL-17A-mediated DVSMC activation was investigated and validated by specific signalling pathway inhibitor.

**Results:**

IL-17A and serum from patients with SSc could promote the proliferation, collagen synthesis and secretion, and migration of DVSMCs. IL-17A neutralising antibody could inhibit the IL-17A-induced activation of DVSMCs. Additionally, IL-17A induced the activation of extracellular-regulated protein kinases 1/2 (ERK1/2) in DVSMCs, and ERK1/2 inhibitor could block the IL-17A-elicited activation of DVSMCs.

**Conclusions:**

Our results suggested that IL-17A derived from patients with SSc might induce the proliferation, collagen synthesis and secretion, and migration of DVSMCs via ERK1/2 signalling pathway, raising the likelihood that IL-17A and ERK1/2 might be promising therapeutic targets for the treatment of SSc-related vasculopathy.

**Electronic supplementary material:**

The online version of this article (doi:10.1186/s13075-014-0512-2) contains supplementary material, which is available to authorized users.

## Introduction

Systemic sclerosis (SSc) is a serious connective tissue disease with unestablished aetiology. The pathogenesis of SSc is microangiopathy, increasing collagen synthesis of fibroblasts and immunological abnormality [1]. The major clinical manifestations are local or systemic sclerosis of skin and visceral organs, leading to high morbidity and mortality rates [2]. According to the scope of the disease, SSc can be divided into two types: diffuse cutaneous SSc (dcSSc) and limited cutaneous SSc (lcSSc) [3].

Microangiopathy is one of the earliest clinical manifestations of patients with SSc, which exists throughout the course of the disease [4]. Raynaud’s phenomenon, digital ulcers, pulmonary arterial hypertension and renal crisis are microangiopathy-related symptoms [5,6]. The major pathological feature of SSc is proliferative or obstructive vascular abnormality, involving the small arteries and arterioles in the skin, lungs, heart and kidneys [7]. In the early stage of skin lesions, the infiltrating inflammatory cells appear primarily in the perivascular region of the dermis layer, including CD4^+^ T cells and monocytes [8]. The histopathology shows vascular wall fibrosis after the early inflammatory injury, manifesting as proliferative vascular endothelial cells and smooth muscle cells, in addition to vascular wall thickening known as ‘onion skin’ [9,10].

T helper 17 (Th17) cells, a CD4^+^ T effector cell type, are characterised by the predominant production of interleukin 17A (IL-17A). Recent studies suggested that Th17 cells and Th17-associated cytokines are involved in SSc [11-13]. Our previous studies have shown an increase of IL-17A in the active phase of SSc patients. IL-17A can promote collagen secretion of fibroblasts and stimulate the expressions of intercellular adhesion molecule 1 (ICAM-1), vascular adhesion molecule 1 (VCAM-1), chemokine (C-X-C motif) receptor 4 (CXCR-4) and chemokine (C-C motif) ligand 20 (CCL-20) in vascular endothelial cells, resulting in endothelial inflammation in SSc [14,15]. In addition to causing the inflammatory response of endothelial cells, whether IL-17A can act on dermal vascular smooth muscle cells (DVSMCs) in the media of vascular walls has not been established. This study elaborates the effect of IL-17A on SSc patient-derived DVSMCs.

In the present study, we first demonstrated increased proliferation, collagen synthesis and secretion, and migration of DVSMCs after being stimulated by human recombinant IL-17A and SSc serum-derived IL-17A. Furthermore, SSc serum-derived IL-17A induced the activation of extracellular-regulated protein kinases 1/2 (ERK1/2) in DVSMCs and PD 98059 (an ERK1/2 inhibitor) could alleviate IL-17A-induced functional activation of DVSMCs. These data suggested that IL-17A and ERK1/2 might play a key role in the pathophysiology of SSc-related microangiopathy.

## Methods

### Patients with SSc and healthy individuals

This study was reviewed and approved by the Zhongshan Hospital Research Ethics Committee; the human study protocol was approved by the Institutional Review Board of Zhongshan Hospital of Fudan University. A total of 16 patients with SSc (n = 6 men and 10 women; mean age, 43.6 ± 8.7 years) were included in the study after they provided their informed consent. All of the patients met the American College of Rheumatology criteria for the classification of SSc [16]. Disease activity was assessed using the criteria proposed by Valentini *et al*. [17], in which the evaluation of clinical and laboratory factors results in a score ranging from 0 to 10 (0 represents no disease activity, and 10 represents maximal activity). All of the enrolled patients exhibited active-stage disease with scores of ≥3, and all of the disease durations were <5 years. All of the patients had different degrees of clinical manifestations of microangiopathy (for example, Raynaud’s phenomenon, digital ulcers, telangiectasia, pulmonary hypertension and renal crisis). In the controls, 15 age- and gender-matched healthy individuals (n = 7 men and 8 women; mean age, 40.7 ± 6.9 years) were enrolled in our study after providing informed consent. Blood samples were obtained from the patients with SSc and healthy individuals. The lesional skin tissues of patients with SSc were obtained to isolate DVSMCs. Two normal skin tissues of age- and gender-matched healthy subjects were obtained to isolate healthy DVSMCs after signing informed consent forms (two tissues from orthopaedic surgery).

### Isolation and identification of SSc patient-derived DVSMCs and healthy subject-derived DVSMCs

The isolation is based on a method reported by Sung Tae Cha *et al*. [18]. We make some changes of the method. Full-thickness 7-mm biopsies of about 50 mm^2^ in size were excised from the hand back lesions of SSc patients and the same area of the controls. After washing the skin with phosphate-buffered solution (PBS) containing 100 U/ml penicillin and 100 μg/ml streptomycin (Invitrogen Corp, Carlsbad, CA, USA) three times for 5 min each, the tissue flap was then incubated for 2 h at 37°C in 5 mg/ml dispase solution (Invitrogen Corp). The dermal blood vessels were isolated under a dissecting microscope (Olympus, Tokyo, Japan). The specimens were cut into 1 mm^3^ cubes and trypsinised with 0.1% trypsin (Life Technologies, Grand Island, NY, USA) for 15 min. Trypsin digestion was terminated using smooth muscle cell medium-basal (Sciencecell, San Diego, CA, USA). After removing the undigested tissues using a 200-mesh filter (Whatman, Maidstone, UK), it was centrifuged for 10 min at 350 × g. The cell pellet was resuspended in 200 μl of PBS and layered onto a 35% Percoll solution (Amersham Biosciences, Uppsala, Sweden). After a continuous density gradient centrifugation for 10 min at 400 × g, the smooth muscle cells have a density greater than 1.065 g/ml. The cell band at the appropriate density was collected and washed in PBS (Invitrogen Corp), it was centrifuged for 10 min at 350 × g. The cell pellet was resuspended using smooth muscle cell medium-basal. The cells from the third to seventh passages were identified as smooth muscle cells using immunofluorescence staining for smooth muscle specific α-actin (α-SMA); smooth muscle myosin heavy chain (myosin) and calponin were positively identified using Western blot. Furthermore, human primary dermal vascular smooth muscle cells, which were purchased from CHI Scientific Inc (Boston, MA, USA), served as a positive control. All cells were cultured at 37°C with 5% CO_2_ and 100% humidity.

### DVSMCs culture and stimulation

To investigate the effect of IL-17A derived from the serum samples of SSc patients on DVSMCs, the cells were seeded in 6-well plates at a density of 3 × 10^5^ cells/well and cultured overnight. Recombinant human IL-17A (eBioscience, San Diego, CA, USA) was added into Dulbecco’s modified Eagle’s medium (DMEM) (Hyclone, Logan City, UT, USA) at concentrations of 1 ng/ml, 10 ng/ml or 100 ng/ml. Five percent of the serum of SSc patients or healthy subjects with or without IL-17A neutralising antibody (8 μg/ml, Abcam, Cambridge, UK) was also added into DMEM. The cells were incubated using IL-17A, 5% SSc serum or healthy serum for 24 h. The proliferation of cells was tested using cell counting kit-8 (CCK-8). The collagen1α1 and collagen3α1 proximal promoter activity were detected using dual-luciferase reporter gene assay (Beyotime, Shanghai, China). The expressions of collagen 1 and collagen 3 were detected using real-time RT-PCR, Western blot and enzyme-linked immunosorbent assay (ELISA). The migration of DVSMCs was detected using transwell assay. For several experiments, DVSMCs were seeded in plates at 90 to 100% confluence and incubated with IL-17A (100 ng/ml) for 0 min, 15 min, 30 min, 45 min and 60 min. The protein of the cells was collected and the phosphorylation status of three major protein kinases (ERK1/2, Jun N-terminal kinase (JNK) and P38) was measured using Western blot. Moreover, the cells were stimulated with 5% SSc serum, 5% healthy serum with or without IL-17A neutralising antibody (8 μg/ml) for 30 min. Subsequently, the phosphorylation of the potential protein kinase was surveyed using Western blot. PD 98059 (Beyotime), a specific inhibitor of ERK1/2 phosphorylation, and SB 203580 (Beyotime), a specific inhibitor of p38-MAP kinase phosphorylation, were used for identifying the downstream signalling mediators of the IL-17 receptor A (IL-17RA. Both PD 98059 and SB 203580 were dissolved in dimethylsulfoxide (DMSO) (Invitrogen Corp) at a concentration of 10 mM and added to the fresh culture medium to achieve the desired final concentrations of 10 μM/ml. The cells were pretreated with PD 98059 or SB 203580 for 2 h and then incubated using IL-17A (100 ng/ml), 5% serum from the patients with SSc or 5% serum from the healthy controls for 24 h.

### CCK-8

According to the manufacturer’s instructions, CCK8 (KeyGEN, Nanjing, Jiangsu, China) was used to measure the proliferation of DVSMCs. First, 2 × 10^3^ cells were plated in a volume of 100 μl into each well of 96-well plates. All assays were performed in quadruplicate. When cells were grown to 90 to 100% confluence, they were given various stimulations for 24 h, 48 h and 72 h. After this incubation period, an orange soluble formazan product formed. The formazan product was spectrometrically quantified using an ELISA reader (Molecular Devices, Sunnyvale, CA, USA) (λ = 450 nm).

### Dual-luciferase reporter assay

The collagen1α1 proximal promoter sequences (containing −174 bp to +42 bp) [19] or collagen3α1 proximal promoter sequences (containing −96 bp to −34 bp) [20] were cloned into pGL3-basic vector (Promega, Madison, WI, USA), leading to the construction of two recombinant plasmids. The inserts were confirmed by DNA sequencing. DVSMCs were transiently transfected at 95% confluence with the recombinant plasmids by Lipofectamine 2000 (Life Technologies), and pRL-SV40 (Promega) was used to normalize the transfection efficiency. Four hours after transfection, the medium was replaced with different concentrations of IL-17A, 5% serum from the patients with SSc or 5% serum from the healthy controls in DMEM. Luciferase activity was tested after 24 h incubation using the dual-luciferase reporter assay system (Beyotime). The ratio of Firefly luciferase to Renilla luciferase activity was calculated to correct transfection efficiency and cell numbers of each well. For each plasmid, three independent transfection experiments were performed, and each was done in triplicate.

### Real-time RT-PCR

Total RNA of DVSMCs was extracted with TRIzol reagent (Life Technologies). Complementary DNA (cDNA) samples were synthesized using the First Strand cDNA Synthesis Kit and oligo(dT) primers (Thermo Fisher Scientific, Waltham, MA, USA). Levels of mRNA for particular genes were examined using SYBR Green PCR Master Mix (Takara, Otsu, Japan). The following primer pairs were used: Hum Collagen 1, forward GTTGTGCGATGACGTGATCTGTGA and reverse TTCTTGGTCGGTGGGTGACTCTG; Hum Collagen 3, forward GCTGGCTACTTCTCGCTCTG and reverse TCCGCATAGGACTGACCAAG; GAPDH, forward GGGGCTCTCCAGAACATCATCC and reverse ACGCCTGCTTCACCACCTTCTT.The 2^-ΔΔCt^ method was used to normalise transcription to GAPDH and to calculate the fold induction relative to controls.

### ELISA

The concentrations of collagen 1 and collagen 3 in the culture supernatants of DVSMCs were detected using the ELISA kit (USCN Life Science, Wuhan, Hubei, China) according to the manufacturer^’^s instructions. Standard protein samples and culture supernatants were incubated for 2 h at room temperature. After washing four to six times, each well was incubated using 50 μl primary anti-human collagen 1 and collagen 3 antibodies for 1 h, respectively. After washing as previously, 100 μl enzyme-labelled antibodies were added into each well and incubated for 1 h at room temperature. Subsequently, solutions A and B were mixed 1:1 and added to each well. Half an hour later, the reaction was terminated by 50 μl buffer. Collagen 1 and collagen 3 concentration data were presented as the means ± standard deviation. The assay was performed in triplicate according to the manufacturer^’^s recommendations.

### Western blot

The proteins of DVSMCs were extracted using RIPA lysis buffer (Beyotime), with the protease inhibitor phenylmethanesulfonyl fluoride (Beyotime). The proteins were separated into 8% or 10% SDS-PAGE (Beyotime), and then transferred onto PVDF membranes (EMD Millipore, Billerica, MA, USA). After being blocked with 5% milk for 2 h at room temperature, the membranes were incubated using primary antibodies, including rabbit anti-human collagen 1 (1:1000, Abcam), rabbit anti-human collagen 3 (1:3000, Abcam), rabbit anti-human α-SMA (1:600, Abcam), goat anti-human IL-17RA (1:1000, Abcam), rabbit anti-human p38 (1:1000, Cell Signaling Technology, Boston, MA, USA), rabbit anti-human phospho-p38 MAPK (1:1000, Cell Signaling Technology), rabbit anti-human JNK (1:1000, Cell Signaling Technology), rabbit anti-human phospho-JNK (1:1000, Cell Signaling Technology), rabbit anti-human ERK1/2 (1:1000, Cell Signaling Technology), rabbit anti-human phospho-ERK1/2 (1:1000, Cell Signaling Technology), and rabbit anti-human GAPDH (1:1000, Cell Signaling Technology) overnight at 4°C. The membranes were then washed and incubated with appropriate HRP-conjugated secondary antibodies for 1.5 h at room temperature. The proteins were detected using ECL detection reagents (Beyotime).

### Transwell

The migration assay of DVSMCs was performed using polycarbonate membrane transwell inserts (8 μm pore size) (Corning, New York, NY, USA). The cells that exhibited growth arrest for 48 h were harvested with 0.05% trypsin-EDTA (Life Technologies) and resuspended (10 × 10^5^ cells/ml) with serum-free DMEM. The cells were added into the upper chamber. IL-17A (100 ng/ml), 5% healthy serum, 5% SSc patient-derived serum with or without neutralising antibody, PD9 8059 or SB 203580 was added into the lower chamber. The cells were incubated for 6 h at 37°C and 5% CO_2_. After scraping the cells affixed to the inside of the membrane, the cells that had migrated through the insert were fixed with 4% paraformaldehyde (Beyotime) and stained with Giemsa (Jiancheng Bioengineering Research Institute, Nanjing, Jiangsu, China) according to the manufacturer^’^s instructions. For each well, the number of migrated cells was counted from five high-power fields (HPF) at 200 × magnification.

### Statistical analysis

The quantitative data are expressed as the means ± standard deviation. Statistical analysis was performed using SPSS 17.0 statistical software (SPSS Inc., Chicago, IL, USA). Statistical significance was determined using analysis of variance followed by Student’s *t* test for comparisons of multiple means. The results showing *P* <0.05 were considered to be statistically significant.

## Results

### IL-17A promotes the proliferation of SSc patient-derived DVSMCs

**Figure 1:**
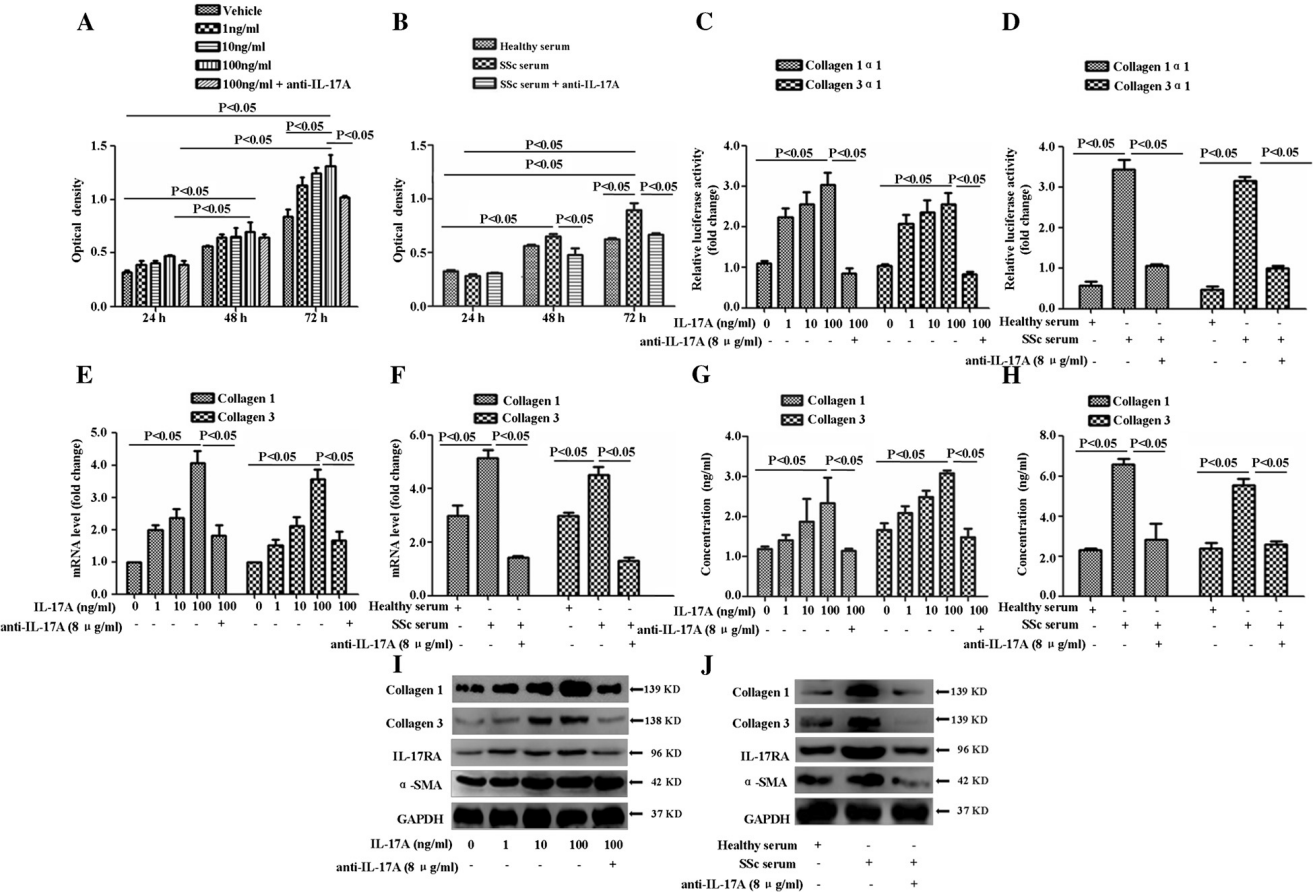
**IL-17A derived from SSc patient serum promotes the proliferation, collagen synthesis and secretion of SSc patient-derived DVSMCs. (A)** DVSMCs were treated with different doses of IL-17A for 24 h, 48 h, 72 h, and cell proliferation was tested using cell counting kit-8. **(B)** The cells were treated with the serum of SSc patients for 24 h, 48 h, 72 h, and cell proliferation was tested using CCK8. **(C)** The activity of collagen1α1 or collagen3α1 proximal promoter was test using a dual luciferase reporter gene assay after being treated with different doses of IL-17A for 24 h. **(D)** The activity of collagen1α1 or collagen3α1 proximal promoter was also detected after being treated with the serum of SSc patients and healthy subject for 24 h. Data were represented as mean ratios of Firefly to Renilla luciferase activity. **(E)** The cells were cultured in the indicated doses of IL-17A for 24 h, the gene expression of collagen 1 and collagen 3 was measured using real-time RT-PCR analysis. **(F)** The cells were stimulated with the serum of SSc patients and healthy subjects for 24 h, and the gene expression of collagen 1 and collagen 3 was measured using real-time RT-PCR analysis. **(G)** The cells were treated with different doses of IL-17A for 24 h, the concentration of collagen 1, collagen 3 was detected using ELISA. **(H)** The cells were treated with the serum of SSc patients and healthy subjects for 24 h, and the concentration of collagen 1, collagen 3 was also detected by ELISA. **(I, J)** The cells were treated with different doses of IL-17A, serum of SSc patients or healthy individuals for 24 h, and the expressions of collagen 1, collagen 3, IL-17RA and α-SMA were measured using Western blot. GAPDH was used as a loading control. The experiment was repeated three times, and the data are presented as means ± standard deviation.

Previous studies have demonstrated thickened dermal vascular walls in the histopathology of systemic scleroderma [21,22]. In the media of the vascular walls, smooth muscle cells apparently proliferate. Our recent study observed that IL-17^+^ lymphocytes infiltrated the vascular walls and perivascular regions, especially in the dermis layer [15]. To investigate the effect of IL-17A on the proliferation of SSc patient-derived DVSMCs, DVSMCs were first isolated from the skin of SSc patients and healthy donors. The expressions of myosin, calponin, α-SMA and IL-17RA were detected using the Western blot or immunofluorescence (See Additional file 1: Figure S1A, B; Additional file 2: Figure S2A-E). The cells were treated with different concentrations of IL-17A for 24 h, 48 h and 72 h. We observed a significant proliferation of SSc patient-derived DVSMCs after incubation with IL-17A (100 ng/ml) for 72 h compared with the vehicle (Figure 1A). A similar proliferative response was observed in the cells incubating with 5% SSc serum (Figure 1B) for 72 h. Pretreatment using IL-17 neutralising antibody, but not its isotype control, inhibited IL-17A-induced proliferation of DVSMCs (Figure 1A,B). Altogether, our results suggested that IL-17A derived from the serum of patients with SSc might stimulate the proliferation of SSc patient-derived DVSMCs.

### IL-17A induces the collagen synthesis and secretion of SSc patient-derived DVSMCs

We investigated the role of IL-17A on the collagen 1 and collagen 3 synthesis and secretion of SSc patient-derived DVSMCs, which are the major components of the vascular wall media. In Th17 cytokine families, IL-17A is identified as the key cytokine associated with autoimmunity, inflammation and host defence [23]. However, it is unknown whether IL-17A can promote collagen 1 and collagen 3 synthesis and secretion of SSc patient-derived DVSMCs. In the present study, we first examined the effect of IL-17A on collagen1α1 and collagen3α1 proximal promoter activity by construction of reporter gene plasmids and dual-luciferase reporter gene assay. The results showed that collagen1α1 and collagen3α1 proximal promoter activity significantly enhanced after being stimulated by human recombinant IL-17A (Figure 1C) and SSc serum-derived IL-17A (Figure 1D). Second, the mRNA of collagen 1 and collagen 3 were also increased after being incubated with IL-17A (Figure 1E) and SSc serum-derived IL-17A (Figure 1F). Third, when IL-17A alone was used to stimulate SSc patient-derived DVSMCs, Western blot demonstrated that the expressions of collagen 1, collagen 3 and α-SMA were increased (Figure 1C). Furthermore, it also showed that 5% serum of SSc patients promoted the expressions of collagen 1, collagen 3 and α-SMA of DVSMCs, and the neutralisation of IL-17A in the culture medium suppressed the expressions of these proteins (Figure 1D). Additionally, we observed that the secretions of collagen 1 and collagen 3 were increased when SSc patient-derived DVSMCs were incubated with 5% SSc serum and different doses of IL-17A (Figure 1E,F). The IL-17A neutralising antibody counteracted the effect of IL-17A on SSc patient-derived DVSMCs (Figure 1E,F). Altogether, these results implied that IL-17A derived from the serum of SSc patients might have a potential role in inducing collagen synthesis and secretion of SSc patient-derived DVSMCs.

### IL-17A stimulates the activation of SSc patient-derived DVSMCs via ERK phosphorylation

**Figure 2:**
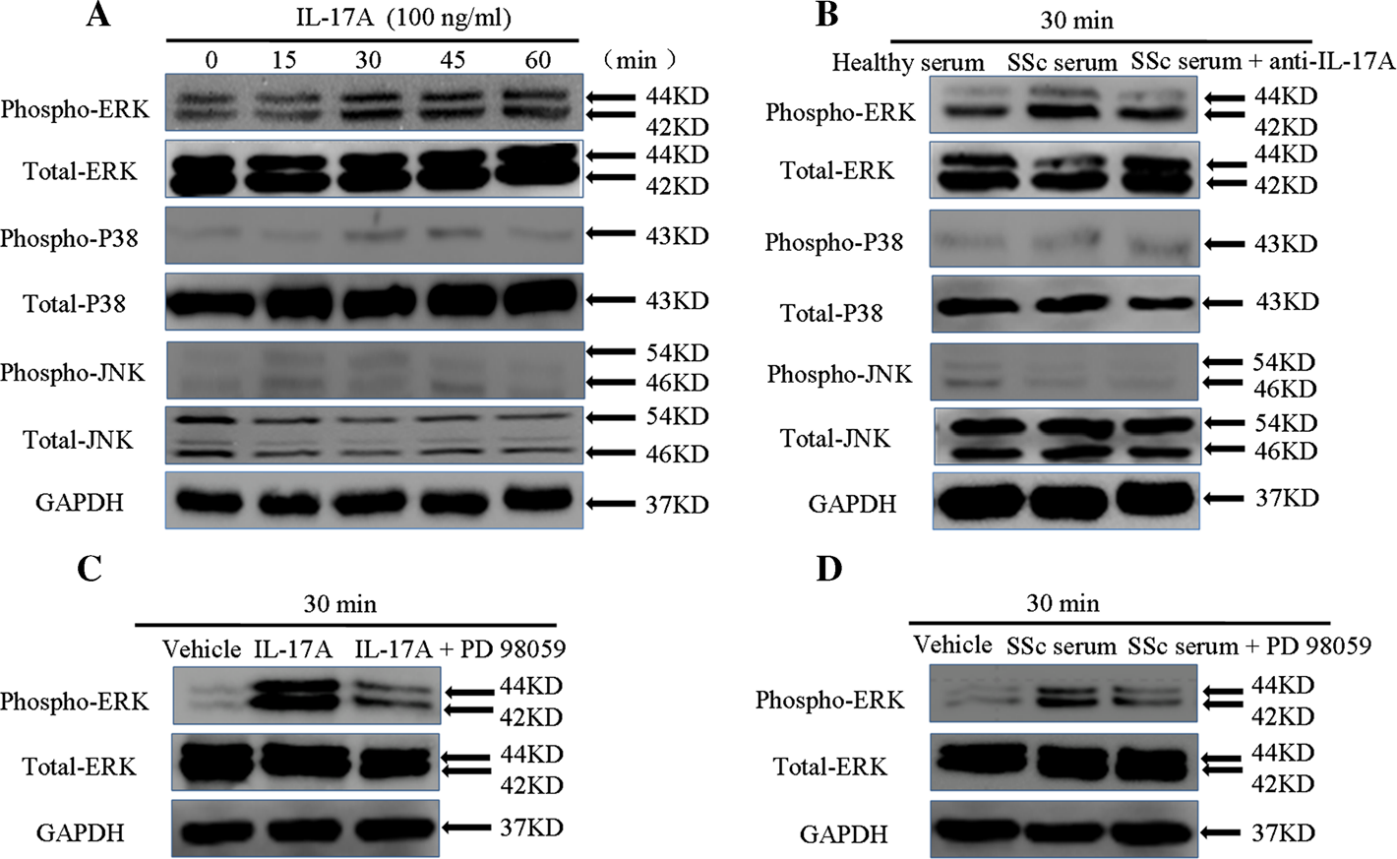
**IL-17A induces ERK1/2 MAPK phosphorylation in SSc patient-derived DVSMCs. (A)** SSc patient-derived DVSMCs were treated with 100 ng/ml IL-17A for 15, 30, 45 and 60 min, and the phosphorylation of ERK1/2, p38 MAPK and JNK were detected using Western blot. **(B)** The cells were exposed to the serum of SSc patients or healthy controls for 30 min, the phosphorylation of ERK1/2, p38 MAPK and JNK were detected by Western blot. **(C)** The cells were incubated with IL-17A (100 ng/ml) in the presence of PD98059 (10 μM/ml) for 30 min, the phosphorylation of ERK1/2 was detected using Western blot. **(D)** The cells were incubated with 5% SSc serum or healthy serum in the presence of PD98059 (10 μM/ml) for 30 min, the phosphorylation of ERK1/2 was detected using Western blot. GAPDH was used as a loading control. The experiment was repeated three times. DVSMCs, dermal vascular smooth muscle cells; ELISA, enzyme-linked immunosorbent assay; ERK, extracellular-regulated protein kinases; IL-17A, interleukin-17A; JNK, Jun N-terminal kinase; MAPK, mitogen-activated protein kinases; SSc, systemic sclerosis.

The results of the above experiments show that IL-17A derived from SSc serum promotes the proliferation, collagen synthesis and secretion of SSc patient-derived DVSMCs; however, the precise mechanisms underlying these effects have not been established. According to the literature, the mitogen-activated protein kinase (MAPK) signalling pathway may be involved in IL-17-mediated inflammation of coronary smooth muscle cells and human umbilical vein endothelial cells [15,24]. To explore the potential involvement of protein kinase pathways in IL-17A-mediated proliferation, collagen synthesis and secretion of SSc patient-derived DVSMCs, we detected phospho-JNK, phospho-p38 MAPK and phospho-ERK1/2 in these cells using Western blot. A prominent increase of phospho-ERK1/2 MAPK level was observed in SSc patient-derived DVSMCs after stimulation with IL-17A (100 ng/ml) (Figure 2A). Additionally, the increased expression of phospho-ERK1/2 was detected when cells were exposed to serum from patients with SSc. Moreover, IL-17 neutralising antibody and PD 98059 (ERK1/2 inhibitor) could weaken the expression of phospho-ERK1/2 MAPK (Figure 2B-D). These experiments revealed that the ERK1/2 MAPK might be an important signalling pathway in IL-17A-induced activation of DVSMCs.

**Figure 3:**
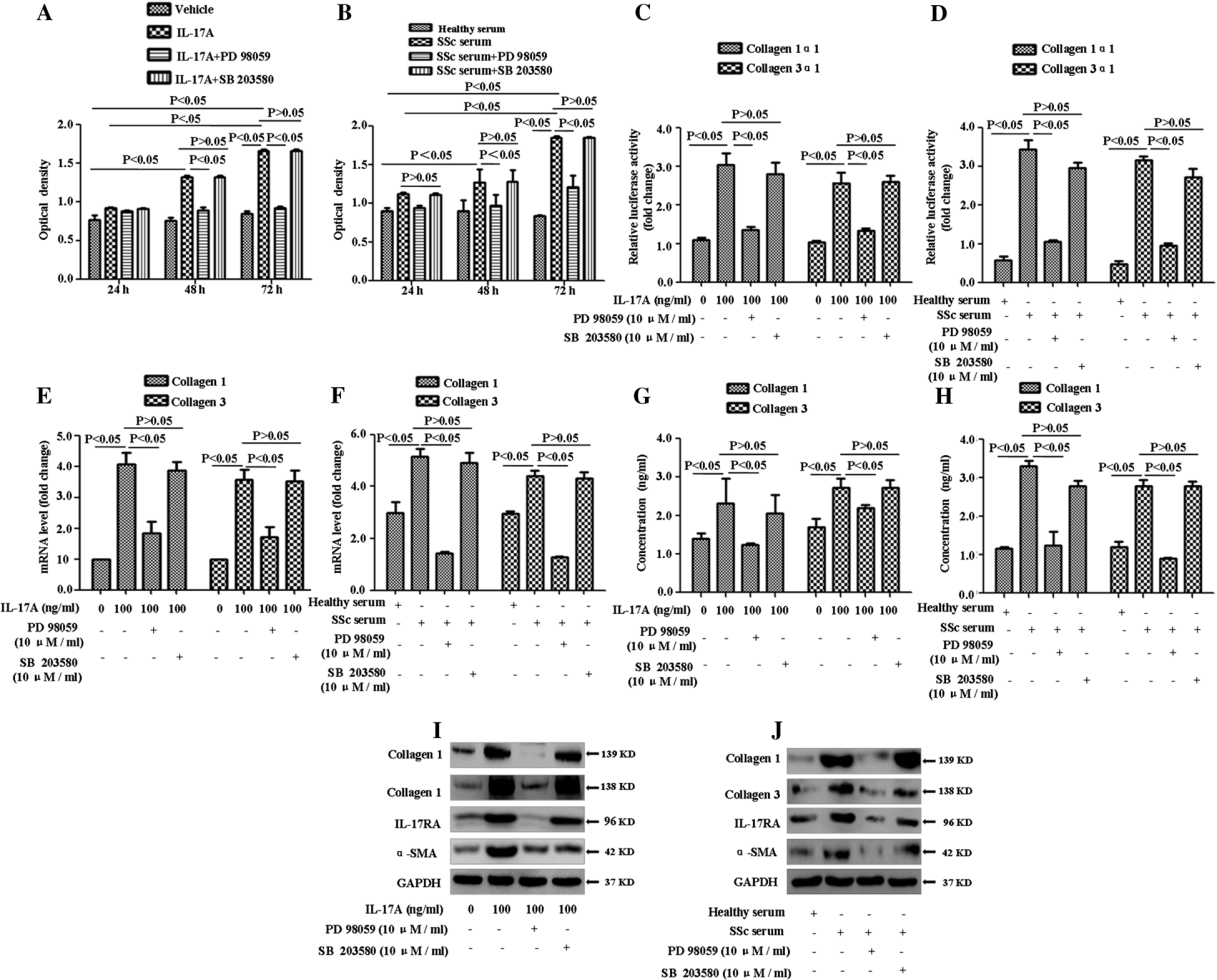
**IL-17A induces proliferation, collagen synthesis and secretion of SSc patient-derived DVSMCs via ERK1/2 signing pathway. (A)** DVSMCs were pre-treated with PD 98059 (10 μM/ml) for 2 h before incubation with IL-17A at 100 ng/ml for 24 h, 48 h, 72 h, and cell proliferation was tested using cell counting kit-8. **(B)** The cells were pre-treated with PD98059 for 2 h before incubation with the serum of SSc patients or healthy individuals for 24 h, 48 h, 72 h, and cell proliferation was tested using CCK8. **(C)** The cells were pre-treated with PD 98059 for 2 h before incubation with IL-17A at 100 ng/ml for 24 h. The activity of collagen1α1 or collagen3α1 proximal promoter was detected using a dual luciferase reporter gene assay. **(D)** After being pre-treated with PD98059 for 2h before incubation with the serum of SSc patients or healthy individuals for 24 h, the activity of collagen1α1 or collagen3α1 proximal promoter was also detected. Data were represented as mean ratios of Firefly to Renilla luciferase activity. **(E, F)** The cells were pre-treated with PD 98059 for 2 h before incubation with IL-17A or SSc serum for 24 h, the gene expression of collagen 1 and collagen 3 was measured using real-time RT-PCR analysis. **(G, H)** The cells were pre-treated with PD 98059 for 2 h before incubation with IL-17A or SSc serum for 24 h, the concentration of collagen 1, collagen 3 was detected using ELISA. **(I, J)** The cells were pre-treated with PD98059 for 2 h before incubation with IL-17A, serum from SSc patients and healthy individuals for 24 h, and the protein expressions of collagen 1, collagen 3, IL-17RA and α-SMA were measured using Western blot. GAPDH was used as a loading control. The experiment was repeated three times, and the data are presented as means ± standard deviation.

To elucidate the role of the ERK1/2, PD 98059 (ERK1/2 inhibitor) and SB 203580 (P38 inhibitor) were used to treat SSc patient-derived DVSMCs in the presence of IL-17A or serum from patients with SSc. PD98059 significantly attenuated IL-17A-mediated upregulation of proliferation, collagen synthesis and secretion of these cells (Figure 3A-J). These results indicated that ERK1/2 signalling pathway might play a key role in IL-17A-induced proliferation, collagen synthesis and secretion of SSc patient-derived DVSMCs.

### IL-17A induces the migration of SSc patient-derived DVSMCs

**Figure 4:**
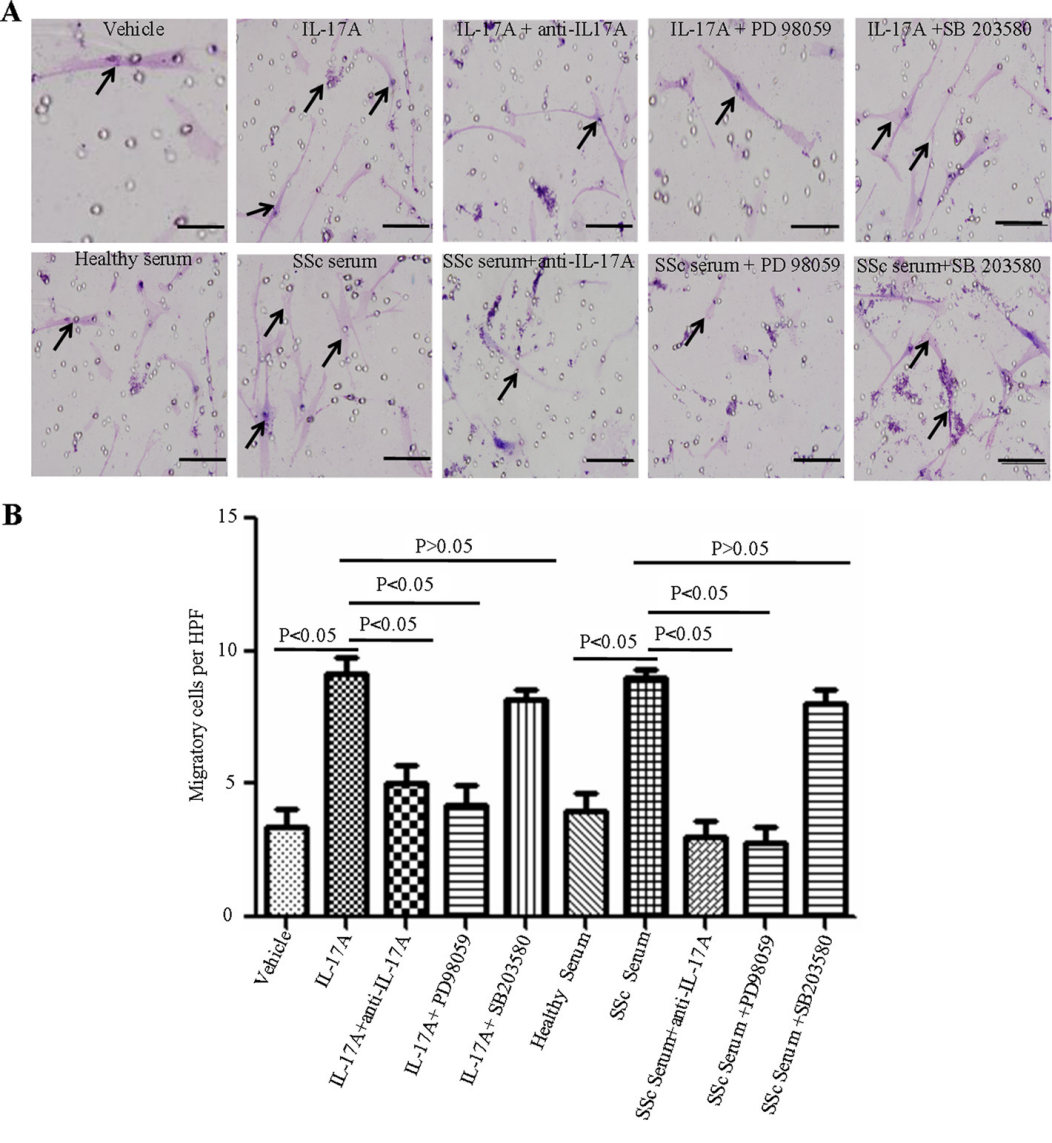
**IL-17A promotes migration of SSc patient-derived DVSMCs by ERK signalling pathway. (A)** SSc patient-derived DVSMCs were incubated with IL-17A (100 ng/ml), 5% healthy serum, 5% SSc serum with or without anti-IL-17A antibody (8 μg/ml), ERK inhibitor (PD98059, 10 μM/ml) or P38 inhibitor SB 203580 (10 μM/ml) for 24 h. Black arrows represent migrated cells in per high-power field (HPF) at 200 × magnification. Scale bar = 100 μm. **(B)** The number of migrated cells in per HPF. The experiment was repeated three times, and the data are presented as mean ± standard deviation. DVSMCs, dermal vascular smooth muscle cells; ELISA, enzyme-linked immunosorbent assay; ERK, extracellular-regulated protein kinases; IL-17A, interleukin-17A; SSc, systemic sclerosis.

Our previous study showed that IL-17A facilitated the adhesion of T cells or peripheral blood mononuclear cells (PBMCs) to vascular endothelial cells by co-culture of Jurkat cells or SSc patient-derived PBMCs with human umbilical vein endothelial cells [15]. Cell adhesion and migration were two interrelated biological processes. In the present study, we investigated the effect of IL-17A on the migration of SSc patient-derived DVSMCs. We found that after stimulation by IL-17A (100 ng/ml) and 5% SSc serum, the number of migrated cells increased significantly (*P* <0.05) compared with negative controls (Figure 4A,B). IL-17A neutralising antibody or PD 98059 reduced the number of migrated cells (*P* <0.05) (Figure 4A,B). These results indicated that IL-17A might promote the migration of SSc patient-derived DVSMCs via the ERK1/2 signalling pathway.

## Discussion

There is growing recognition that Th17 cells are involved in a variety of human diseases, for example, systemic lupus erythematosus (SLE), psoriasis, rheumatoid arthritis, multiple sclerosis, colitis, atherosclerosis, asthma, and particularly autoimmune diseases [25-33]. Moreover, increasing evidence suggests that IL-17-producing cells are related to SSc, in which Th17 cells and characteristic cytokines increase significantly [34]. Furthermore, Th17 cytokines have been observed to be associated with the activity of SSc [14,35]. IL-17A may induce endothelial inflammation in SSc [15]. To the best of our knowledge, there is no research concerning the effect of Th17 cells on SSc patient-derived DVSMCs.

Because of the importance of the Th17 cytokine family in SSc, we aimed to explore the relationship between these cytokines and SSc patient-derived DVSMCs, which was the potential factor for vascular wall thickening. In the present study, we confirmed that Th17 cytokines might promote the proliferation of SSc patient-derived DVSMCs for the first time. IL-17A enhanced the proliferation of SSc patient-derived DVSMCs in a dose- and time-dependent fashion compared with healthy DVSMCs (See Additional file 3: Figure S3A, B). The peak proliferation was at 100 ng/ml within 72 h. These cells also proliferated apparently after being stimulated by SSc serum. IL-17A neutralising antibody inhibited IL-17A-induced proliferation of SSc patient-derived DVSMCs. These results were consistent with other studies concerning asthma, which revealed that Th17-associated cytokines promote human airway smooth muscle cell proliferation [33].

In addition to cell proliferation, we examined IL-17A-mediated collagen synthesis and secretion from SSc patient-derived DVSMCs. We stimulated cells using different concentrations of IL-17A and SSc serum, collagen synthesis and secretion were tested using dual-luciferase reporter assay, real-time RT-PCR, Western blot and ELISA. Collagen 1 and collagen 3 of SSc patient-derived DVSMCs increased at promoter, mRNA and protein levels compared with healthy DVSMCs (See Additional file 3: Figure S3B-J). Moreover, our study showed that IL-17A promoted the migration of SSc patient-derived DVSMCs. We speculated that IL-17A might promote the migration of vascular smooth muscle cells from the vascular media to the intima, resulting in intimal thickening, which is similar to their role in atherosclerosis. The migration of vascular smooth muscle cells is one of the key factors that contribute to atherosclerosis; many growth factors and chemotactic factors are involved in this process.

Mitogen-activated protein kinases (MAPKs), a class of serine/threonine protein kinases, include ERK, c-JNK and p38 MAPK. Several studies have demonstrated that MAPKs signal transduction pathways are present in the majority of cells, inducing cell proliferation, differentiation, transformation and apoptosis [36-38]. Studies have reported that the ERK pathway might be associated with airway smooth muscle cell proliferation of asthma patients [39]. IL-17A promotes the migration of asthma patient-derived smooth muscle cells through p38-MAPK signalling pathways. However, how the signalling pathway of IL-17A induced the functional activation of SSc patient-derived DVSMCs remains unclear. In the present study, we found that the treatment of SSc patient-derived DVSMCs using IL-17A resulted in increased phosphorylation of ERK1/2, but not p38-MAPK or JNK. Additionally, ERK1/2 inhibitor (PD98059) significantly inhibited IL-17A-mediated proliferation, collagen synthesis and secretion, and migration of SSc patient-derived DVSMCs. These data suggest that ERK1/2 phosphorylation may play a key role in IL-17A-induced dysfunction of SSc patient-derived DVSMCs.

One of the advantages of this study is the ability to analyse the effect of IL-17A on SSc patient-derived DVSMCs, which shows that IL-17A derived from the serum of SSc patients has the capability of enhancing proliferation, collagen synthesis and secretion, and migration. This effect may be dependent on the ERK1/2 MAPK signalling pathway.

## Conclusions

Altogether, our data raise the likelihood that Th17 cytokines may play a role in vascular wall remodelling and provide evidence of a new mechanism that may involve vascular fibrosis in patients with SSc. Thus, targeting the control of ERK activation may provide a new therapeutic approach for the treatment of microangiopathy of SSc. However, whether this hypothesis is valid *in vivo* requires additional investigation.

## Additional files

Additional file 1: Figure S1. The morphology and identification of SSc patient-derived DVSMCs. **(A)** The morphology and positive marker (α-SMA, green fluorescence) of SSc patient-derived DVSMCs were shown. **(B)** Myosin, calponin, α-SMA and IL-17RA expression in SSc patient-derived DVSMCs and human primary dermal vascular smooth muscle cells (HDVSMCs) were examined by means of western blot assay. GAPDH was used as a loading control. Additional file 2: Figure S2. The morphology and identification of healthy subject-derived DVSMCs. The morphology and positive markers (myosin, red fluorescence; calponin, green fluorescence) of healthy subject-DVSMCs were shown. Myosin and calponin were detected using double immunofluorescence staining. Additional file 3: Figure S3. The effect of IL-17A on healthy subject-derived DVSMCs. **(A)** Healthy subject-derived DVSMCs were treated with different doses of IL-17A for 24 h, 48 h, 72 h, and cell proliferation was detected using CCK8. **(B)** The cells were treated with the serum of SSc patients and healthy subject for 24 h, 48 h, 72 h, and cell proliferation was detected using CCK8. **(C, D)** A dual luciferase reporter gene assay with a short fragment of the collagen1α1 or collagen3α1 proximal promoter was performed in the cells after being treated with different doses of IL-17A, the serum of SSc patients or healthy subjects for 24 h. **(E, F)** The cells were cultured in the indicated doses of IL-17A, the serum of SSc patients or healthy subjects for 24 h, the gene expression of collagen 1 and collagen 3 was measured using real-time PCR analysis. **(G, H)** The cells were treated with different doses of IL-17A, the serum of SSc patients or healthy subjects for 24 h, and the concentration of collagen 1 and collagen 3 in supernatants was detected using ELISA. **(I, J)** The cells were treated with different doses of IL-17A and serum of SSc patients and healthy individuals for 24 h, and the protein expression of collagen 1, collagen 3 and α-SMA was measured using Western blot. GAPDH was used as a loading control. **(K)** The cells were incubated with IL-17A, healthy serum, SSc serum with or without anti-IL-17A antibody (8 μg/ml) for 24 h. Black arrows represent magrated cells in per high-power field at 200 × magnification. Scale bar = 100 μm. The experiment was repeated three times, and the data are presented as means ± standard deviation.

## Abbreviations

α-SMA: α-smooth muscle actin
CCK-8: cell counting kit-8
CCL-20: chemokine (C-C motif) ligand 20
CXCR-4: chemokine (C-X-C motif) receptor 4
DMEM: Dulbecco’s modified Eagle’s medium
DMSO: dimethyl sulfoxide
dSSc: diffuse cutaneous SSc
DVSMCs: dermal vascular smooth muscle cells
ELISA: enzyme-linked immunosorbent assay
ERK: extracellular-regulated protein kinases
HPF: high-power field
ICAM-1: intercellular adhesion molecule 1
IL-17A: interleukin-17A
IL-17RA: IL-17 receptor A
JNK: Jun N-terminal kinase
lSSc: limited cutaneous SSc
MAPK: mitogen-activated protein kinases
PBMCs: peripheral blood mononuclear cells
PBS: phosphate-buffered saline
SLE: systemic lupus erythematosus
SSc: systemic sclerosis
Th17: T helper 17
VCAM-1: vascular adhesion molecule 1
