# Supplementary figures and images for: Interleukin-17A promotes functional activation of systemic sclerosis patient-derived dermal vascular smooth muscle cells by extracellular-regulated protein kinases signalling pathway
Source: Arthritis Res Ther. 2014 Dec 31;16(6):4223. doi: 10.1186/s13075-014-0512-2 (PMC4316765; doi:10.1186/s13075-014-0512-2)

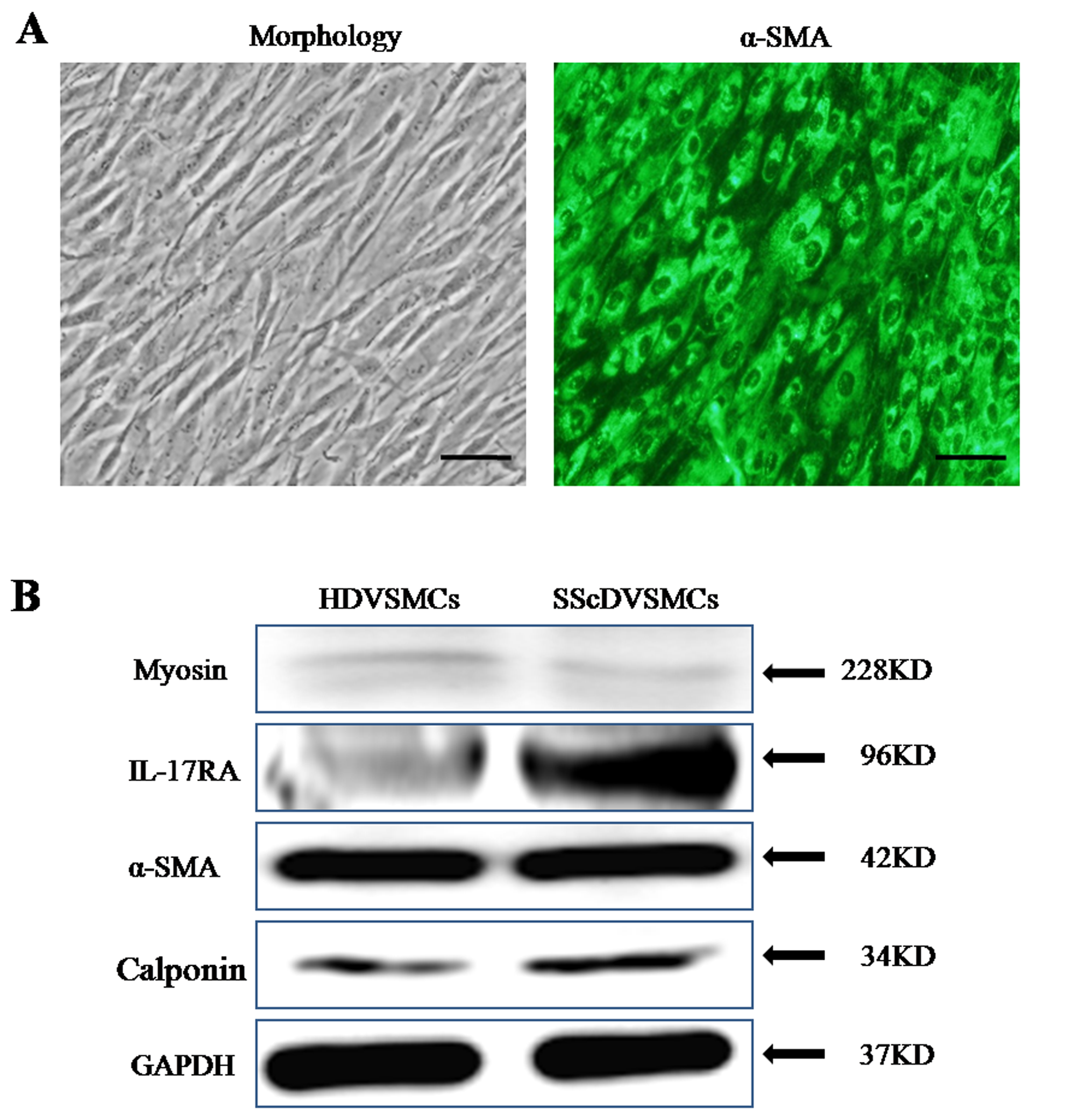

Supplement: Additional file 1: Figure S1. — The morphology and identification of SSc patient-derived DVSMCs. (A) The morphology and positive marker (α-SMA, green fluorescence) of SSc patient-derived DVSMCs were shown. (B) Myosin, calponin, α-SMA and IL-17RA expression in SSc patient-derived DVSMCs and human primary dermal vascular smooth muscle cells (HDVSMCs) were examined by means of western blot assay. GAPDH was used as a loading control. [file 13075_2014_512_MOESM1_ESM.tiff]

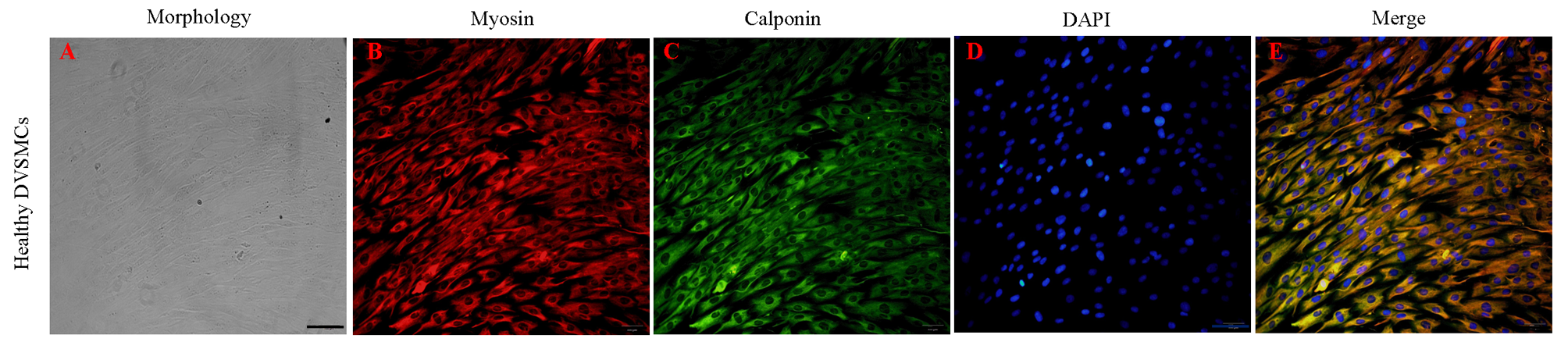

Supplement: Additional file 2: Figure S2. — The morphology and identification of healthy subject-derived DVSMCs. The morphology and positive markers (myosin, red fluorescence; calponin, green fluorescence) of healthy subject-DVSMCs were shown. Myosin and calponin were detected using double immunofluorescence staining. [file 13075_2014_512_MOESM2_ESM.tiff]

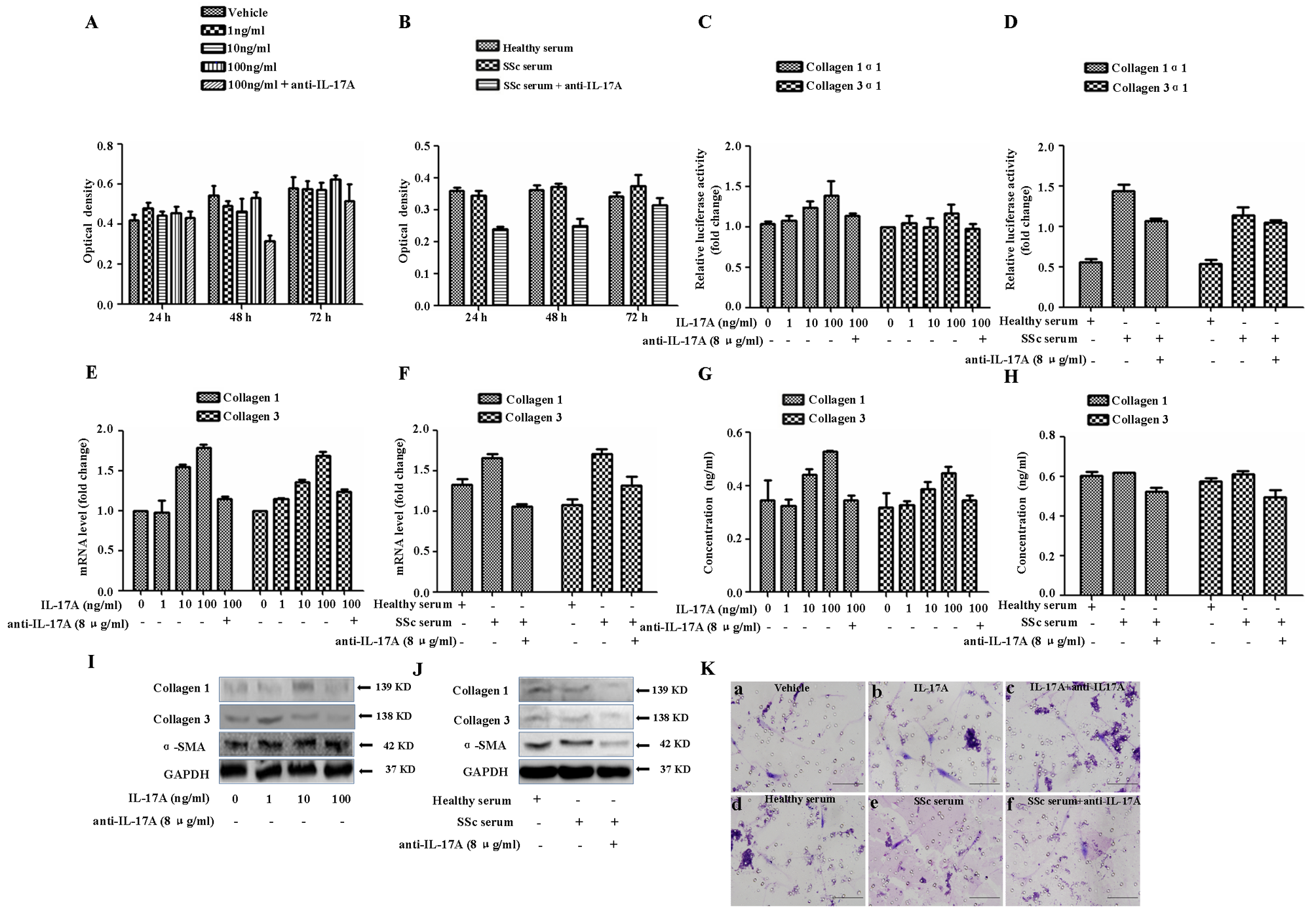

Supplement: Additional file 3: Figure S3. — The effect of IL-17A on healthy subject-derived DVSMCs. (A) Healthy subject-derived DVSMCs were treated with different doses of IL-17A for 24 h, 48 h, 72 h, and cell proliferation was detected using CCK8. (B) The cells were treated with the serum of SSc patients and healthy subject for 24 h, 48 h, 72 h, and cell proliferation was detected using CCK8. (C, D) A dual luciferase reporter gene assay with a short fragment of the collagen1α1 or collagen3α1 proximal promoter was performed in the cells after being treated with different doses of IL-17A, the serum of SSc patients or healthy subjects for 24 h. (E, F) The cells were cultured in the indicated doses of IL-17A, the serum of SSc patients or healthy subjects for 24 h, the gene expression of collagen 1 and collagen 3 was measured using real-time PCR analysis. (G, H) The cells were treated with different doses of IL-17A, the serum of SSc patients or healthy subjects for 24 h, and the concentration of collagen 1 and collagen 3 in supernatants was detected using ELISA. (I, J) The cells were treated with different doses of IL-17A and serum of SSc patients and healthy individuals for 24 h, and the protein expression of collagen 1, collagen 3 and α-SMA was measured using Western blot. GAPDH was used as a loading control. (K) The cells were incubated with IL-17A, healthy serum, SSc serum with or without anti-IL-17A antibody (8 μg/ml) for 24 h. Black arrows represent magrated cells in per high-power field at 200 × magnification. Scale bar = 100 μm. The experiment was repeated three times, and the data are presented as means ± standard deviation. [file 13075_2014_512_MOESM3_ESM.tiff]
